# Comparative Genomics Analyses Reveal the Differences between *B. longum* subsp. *infantis* and *B. longum* subsp. *longum* in Carbohydrate Utilisation, CRISPR-Cas Systems and Bacteriocin Operons

**DOI:** 10.3390/microorganisms9081713

**Published:** 2021-08-11

**Authors:** Mingjie Li, Xingya Zhou, Catherine Stanton, R. Paul Ross, Jianxin Zhao, Hao Zhang, Bo Yang, Wei Chen

**Affiliations:** 1 State Key Laboratory of Food Science and Technology, Jiangnan University, Wuxi 214122, China; limingjie@stu.jiangnan.edu.cn (M.L.); xingyazhouzxy@sina.com (X.Z.); zhaojianxin@jiangnan.edu.cn (J.Z.); zhanghao61@jiangnan.edu.cn (H.Z.); chenwei66@jiangnan.edu.cn (W.C.); 2 School of Food Science and Technology, Jiangnan University, Wuxi 214122, China; 3 International Joint Research Laboratory for Pharmabiotics & Antibiotic Resistance, Jiangnan University, Wuxi 214122, China; catherine.stanton@teagasc.ie (C.S.); p.ross@ucc.ie (R.P.R.); 4 APC Microbiome Ireland, University College Cork, T12 K8AF Cork, Ireland; 5 Teagasc Food Research Centre, Moorepark, Fermoy, P61 C996 Co. Cork, Ireland; 6 National Engineering Research Center for Functional Food, Jiangnan University, Wuxi 214122, China; 7 Wuxi Translational Medicine Research Center and Jiangsu Translational Medicine Research Institute Wuxi Branch, Wuxi 214122, China

**Keywords:** *B. longum* subsp. *infantis*, *B. longum* subsp. *longum*, comparative genomics, carbohydrate metabolism, CRISPR-Cas systems, bacteriocin

## Abstract

*Bifidobacterium longum* is one of the most widely distributed and abundant *Bifidobacterium* in the human intestine, and has been proven to have a variety of physiological functions. In this study, 80 strains of *B. longum* isolated from human subjects were classified into subspecies by ANI and phylogenetic analyses, and the functional genes were compared. The results showed that there were significant differences in carbohydrate metabolism between the two subspecies, which determined their preference for human milk oligosaccharides or plant-derived carbohydrates. The predicted exopolysaccharide (EPS) gene clusters had large variability within species but without difference at the subspecies level. Four subtype CRISPR-Cas systems presented in *B. longum*, while the subtypes I-U and II-C only existed in *B. longum* subsp. *longum*. The bacteriocin operons in *B. longum* subsp. *infantis* were more widely distributed compared with *B. longum* subsp. *longum*. In conclusion, this study revealed the similarities and differences between *B. longum* subsp. *infantis* and *B. longum* subsp. *longum,* which could provide a theoretical basis for further exploring the probiotic characteristics of *B. longum*.

## 1. Introduction

*Bifidobacterium longum* consists of three subspecies, including *B. longum* subsp. *suis*, *B. longum* subsp. *longum* and *B. longum* subsp. *infantis*, which is one of the most abundant *Bifidobacterium* in the intestines [1]. *B. longum* subsp. *longum* is widely present in the human intestine of different ages, while *B. longum* subsp. *infantis* mainly exists in the intestine of breast-fed infants, and *B. longum* subsp. *suis* is mainly isolated from the gastrointestinal tract of piglets or cattle [2]. At present, *B. longum* subsp. *infantis* and *B. longum* subsp. *longum* have been widely used in dairy products, functional foods and probiotic products, and they have various physiological functions such as regulating immunity [3,4] and maintaining intestinal balance [5]. Although the genetic relationship between *B. longum* subsp. *infantis* and *B. longum* subsp. *longum* is extremely similar, there are still some diversities in phenotypic and genetic determinants. For instance, *B. longum* subsp. *infantis* can metabolise human milk oligosaccharides (HMOs), while *B. longum* subsp. *longum* has a preference for plant-derived carbohydrates [6]. In addition, RAPD-PCR, ribose typing and other genotyping techniques, as well as comparative genomic analysis, revealed the differences in genetic determinants between *B. longum* subsp. *infantis* and *B. longum* subsp. *longum* [7].

Comparative genomics analyses could help understand the genomic characteristics of different strains and their relationship with phenotypes, the differences in strains to compete and adapt in the intestine, and the interaction with the hosts. Gene sequencing technology has developed rapidly in recent years, and the publicly available genomes of *B. longum* have continued to increase, providing more data for genomic comparison. Previous studies revealed the general characteristics of *B. longum* genomes, the compositional characteristics of its pan-genome and the differences in the glycosylhydrolase genes of different subspecies. In addition, those studies discussed the similarities and differences of plasmids, CRISPR-Cas systems and adhesion genes among different strains, and expanded the understanding of the intraspecies genomic diversity of *B. longum* [8,9,10,11]. Unfortunately, the strains used in those studies were mainly *B. longum* subsp. *longum*, and only a few *B. longum* subsp. *infantis* genomes have been involved. Hence, the results of those studies could not fully reflect the characteristics of *B. longum* subsp. *infantis* genomes and the diversities in genetic information among *B. longum* subsp. *infantis* strains.

In this study, *B. longum* were identified at the subspecies level based on ANI and phylogenetic analysis. The similarities and differences of genomes between *B. longum* subsp. *infantis* and *B. longum* subsp. *longum* were explored by the comparative genomics approach, including pan-genome, subspecies specific gene, carbohydrate utilisation, exopolysaccharide (EPS) gene clusters, CRISPR-Cas systems and bacteriocin operons. 

## 2. Materials and Methods

### 2.1. B. Longum Strain, Genonic Sequencing and Data Assembly

The 40 *B. longum* strains used in this study were isolated from human faeces and preserved in our lab (Table 1). All the strains were cultured anaerobically in de Man, Rogosa and Sharpe plus 0.05% (*w/v*) L-cysteine hydrochloride (mMRS) medium at 37 °C. The Illumina Hiseq × 10 platform (Majorbio BioTech Co, Shanghai, China) was used to sequence the draft genomes, SOAPdenovo v2.04 was used to assemble the reads and GapCloser v1.12 was used to fill the local inner gaps referred to previous research [12]. In this study, the genomes of *B. longum* subsp. *infantis* ATCC15697, *B. longum* subsp. *longum* NCC2705 and 38 other available strains randomly selected in the NCBI RefSeq database (including 19 *B. longum* subsp. *infantis* strains and 19 *B. longum* subsp. *longum* strains) were used for genomic comparison (Table 1). In addition, *B. longum* subsp. *infantis* ATCC15697 was acquired from China General Microbiological Culture Collection Centre.

### 2.2. Average Nucleotide Identity (ANI) Values

A python script [13] (https://github.com/widdowquinn/pyani) (accessed on 20 July 2021) was used to calculate the ANI values between each two genomes, and TBtools was used to cluster and visualise the resulting matrix [14]. Ten genomes of *B. longum* subsp. *suis* in the NCBI RefSeq database were supplemented for calculating ANI (Table S1).

### 2.3. Phylogenetic Analyses

The orthologous gene analysis was carried out using orthomclV2.0.9 software [15], the mafft-7.313 [16] was used to align the orthologous gene sequences of different strains and phylogeny software was used to analyse the evolutionary relationship via the neighbour-joining (NJ) method and construct a phylogenetic tree. The optimization of the phylogenetic tree was completed on the online website (http://www.evolgenius.info/evolview/) (accessed on 15 December 2020) [17].

### 2.4. Pan-Genome and Core-Genome Analysis

The pan-genome and the function of core genes were analysed via PGAP v1.2.1 [18], and functional classification of core genes was based on the COG database.

### 2.5. Whole Genome and Orthologous Gene Comparison

The sequence similarities of whole genomes among different strains in the same subspecies were visualised by BLAST Ring Image Generator (BRIG) [19]. OrthomclV2.0.9 software [15] was used to analyse the orthologous genes of *B. longum* subsp. *infantis* and *B. longum* subsp. *longum* and to compare the similarities and differences between the two subspecies.

### 2.6. Genotype and Phenotype Analysis of Carbohydrate Metabolism

The HMM method in HMMER-3.1 was used to annotate all the genomes. The CAZy database was used to predict the carbohydrate active enzyme genes [20], and BLAST was used to compare the carbohydrate metabolism gene clusters. The growth of 41 *B. longum* strains on the medium with six carbohydrates as the sole carbon source was determined, including L-arabinose, L-fucose, lacto-N-tetraose (LNT), 2′-fucosyllactose (2′FL), 3′-sialyllactose (3′SL) and galactooligosaccharide (GOS). Glucose-free mMRS medium with bromocresol purple as an indicator was prepared and the carbohydrate solution filtered through a 0.22-µm sterile membrane filter was added to the glucose-free mMRS medium at a final concentration of 1% (*w/v*). After 48 h anaerobic culturing at 37 °C, a colour change of the medium was observed, and the experiment was repeated independently three times.

### 2.7. CRISPR-Cas Systems Prediction

CRISPRCasFinder was used to predict the CRISPR systems, together with *cas* genes [21]. A phylogenetic tree based on Cas1 protein amino acid sequences and repeat nucleic acid sequences was constructed via MEGA X [22], and sequence alignment was performed using MUSCLE and UPGMA methods to construct a phylogenetic tree. The conserved repeat sequence secondary structure was visualised via RNAfold [23], and Weblogo was used to predict the conservation of RNA secondary structure [24]. The potential prophages in *B. longum* were predicted by PHASTER [25], and a local BLAST database was built based on the prophage sequences to analyse the match between CRISPR spacers and the prophages. 

### 2.8. Bacteriocin Prediction

The online database BAGEL4 was used to predict the potential bacteriocins in *B. longum* [26]. Core peptide BLAST was performed to confirm the bacteriocins identified by BAGEL4.

### 2.9. Statistical Analysis

The number of GH genes, GT genes and CRISPR spacers in *B. longum* genomes were statistically analysed. GraphPad Prism 9.0 (GraphPad Software Inc., San Diego, CA, USA) was utilised for data analysis and plotting, and the significant difference of gene number was evaluated by *t* test.

## 3. Results

### 3.1. ANI and Phylogenetic Analysis of B. longum

**Figure 1:**
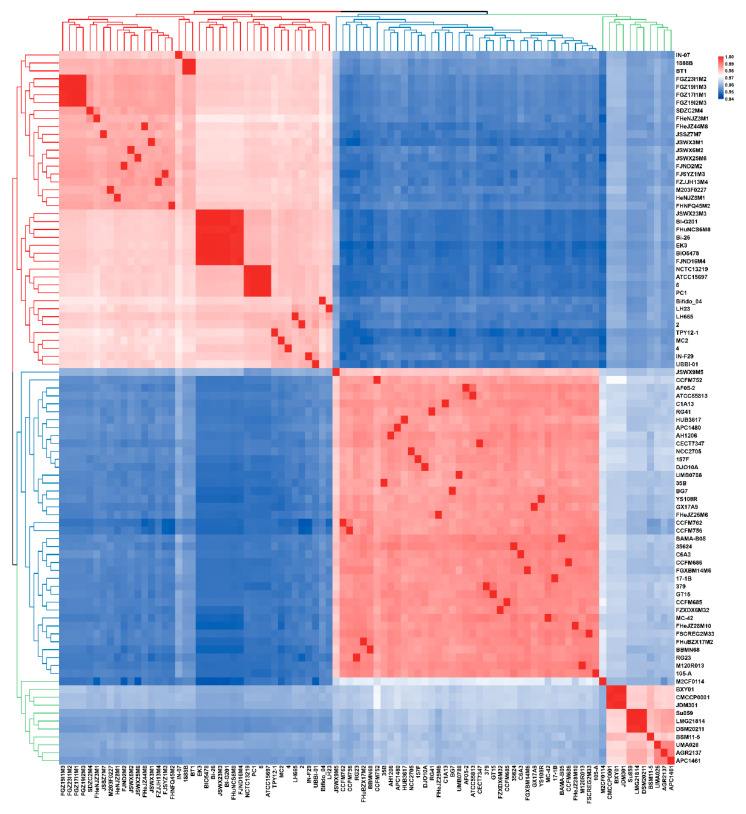
Heatmap based on average nucleotide identity (ANI) of *B. longum*. The gradation of colour from blue to white to red represents an increasing ANI value. The blue branch represents *B. longum* subsp. *longum*, the red branch represents *B. longum* subsp. *infantis* and the green branch represents *B. longum* subsp. *suis.*

ANI is the average of all orthologous genes consistency in the two genomes, which can be used for species classification. As 16S rRNA gene comparison analysis cannot accurately distinguish the subspecies, all the *B. longum* strains in the study were classified at the subspecies level via ANI and phylogenetic analysis. The clustering results based on ANI values showed that 90 strains could be divided into three subspecies (Figure 1, Table S2). The mean of ANI value between *B. longum* subsp. *infantis* and *B. longum* subsp. *longum* was 95.21%, while the mean of ANI between *B. longum* subsp. *suis* and *B. longum* subsp. *infantis* or *B. longum* subsp. *longum* were 95.80% and 96.29%, respectively. Moreover, the mean of ANI between *B. longum* M2CF0114 and *B. longum* subsp. *longum* or *B. longum* subsp. *suis* were 96.90% and 95.97%, respectively. Therefore, *B. longum* M2CF0114 should be classified as *B. longum* subsp. *longum*, although the clustering of ANI puts it in the same branch as *B. longum* subsp. *suis*.

**Figure 2:**
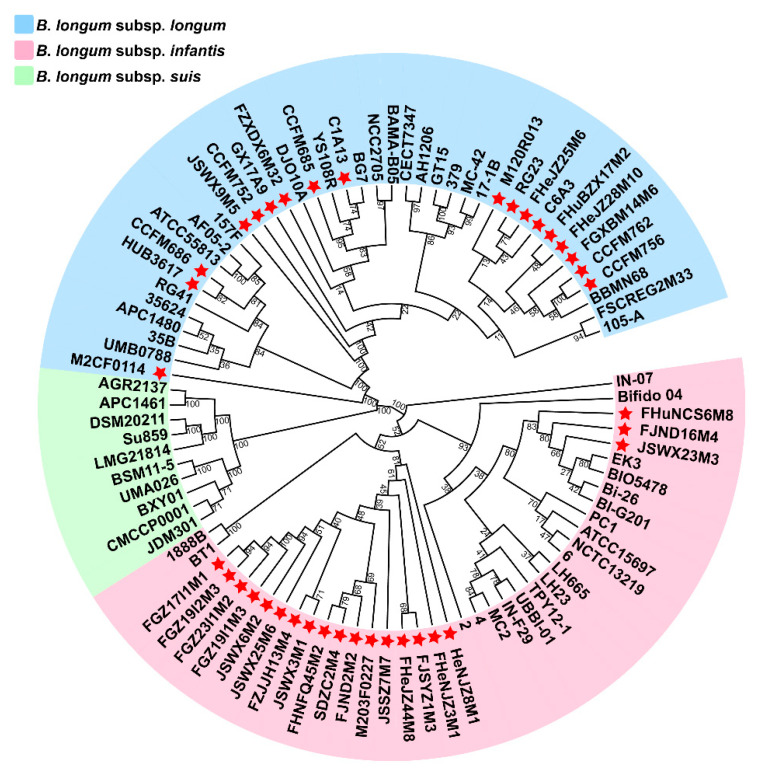
Phylogenetic tree of *B. longum* based on orthologous genes. The blue area represents *B. longum* subsp. *longum*, the red area represents *B. longum* subsp. *infantis* and the green area represents *B. longum* subsp. *suis.* The red stars represent the strains previously isolated from human faeces samples in our lab. Bootstrap values are shown on each node.

Phylogenetic analysis was performed to further confirm the classification of the strains. OrthoMCL was used to analyse the orthologous genes of 90 *B. longum* strains; a total of 849 orthologous genes were obtained, and a phylogenetic tree of *B. longum* was constructed based on the orthologous genes (Figure 2). The results showed that 90 strains were located on three branches, 39 strains were located on the same branch with *B. longum* subsp. *longum* NCC2705 (type strain) and 39 strains were located on the same branch with *B. longum* subsp. *infantis* ATCC15697 (type strain); *B. longum* Su859 and LMG 21814 among the 10 strains on the third branch have been proved to be *B. longum* subsp. *suis* [2,27]. Moreover, it was further confirmed that M2CF0114 belonged to *B. longum* subsp. *longum*. In addition, those analyses showed that some strains were misclassified in the previous study; for instance, 157F, ATCC55813 and 35624 should be *B. longum* subsp. *longum* instead of *B. longum* subsp. *infantis*, and BXY01, CMCCP0001 and JDM301 should be classified as *B. longum* subsp. *suis* rather than *B. longum* subsp. *longum*.

### 3.2. General Genome Features of B. longum subsp. infantis and B. longum subsp. longum 

The general genome features of *B. longum* are shown in Table 1. The genome sizes of *B. longum* subsp. *longum* strains ranged from 2.20 Mb (M120R013) to 2.54 Mb (JSWX9M5), with an average size of 2.39 Mb. The average size of *B. longum* subsp. *infantis* genomes was 2.66 Mb, among which FJND2M2 represented the smallest genome (2.54 Mb) and FHNFQ45M2 possessed largest genome with a size of 2.84 Mb. The average GC content of *B. longum* subsp. *longum* was 60.01%, ranging from 59.69% of CCFM685 to 60.40% of AF05-2, and the average number of genes was 1978. While the average GC content of *B. longum* subsp. *infantis* was 59.56%, and the number of genes ranged from 2011 to 2781, with an average of 2315.

### 3.3. Pan- and Core-Genome of B. longum subsp. infantis and B. longum subsp. longum 

**Figure 3:**
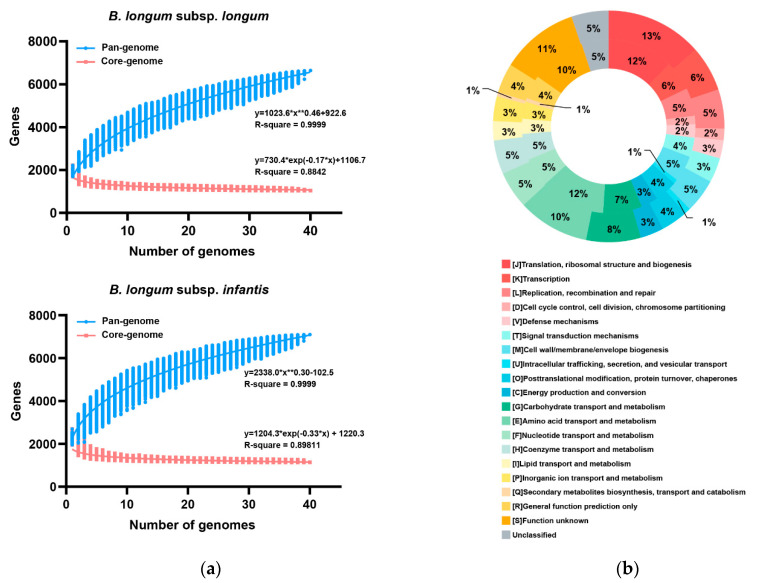
Pan-genome and core-genome of *B. longum* subsp. *longum* and *B. longum* subsp. *infantis*: (**a**) Numbers of pan-genes (blue) and core-genes (red) as a function of the number of genomes; * multiplication, ** exponentiation (**b**) Comparison of core gene function based on the COG database between *B. longum* subsp. *longum* (outside ring) and *B. longum* subsp. *infantis* (inside ring).

Pan-genome refers to all the genes of one species, consisting of three parts: core genes, non-essential genes and strain-specific genes. The pan-genome analysis of *B. longum* subsp. *longum* showed that as the number of added genomes increased, new genes continued to appear, and the number of pan-genome gradually increased; when the number of genomes reached 35, the number of pan-genomic genes increased slowly (Figure 3a), which indicated that the *B. longum* subsp. *longum* genome was nearly closed. The pan-genome of *B. longum* subsp. *longum* contained 6645 genes, and its core genome possessed 1043 genes. The COG annotation showed that those genes related to translation, ribosomal structure and biogenesis accounted for the highest proportion in the core genome (13.42%), followed by function unknown (10.83%), amino acid transport and metabolism related genes (9.97%), and the genes related to carbohydrate transport and metabolism accounted for 7.57% in the core genome (Figure 3b). The pan-genome curve of *B. longum* subsp. *infantis* showed that it was approximately closed; as the number of added genomes increased, the rate of change in the number of core genomes slowed down (Figure 3a). The core genome size of *B. longum* subsp. *infantis* strains was 1139 genes, which accounted for approximately 16.06% of the *B. longum* subsp. *infantis* pan-genome. The functional composition of the COG of *B. longum* subsp. *infantis* was not significantly different from that in *B. longum* subsp. *longum* (Figure 3b), and only slightly higher than *B. longum* subsp. *longum* (12.20%) in genes related to amino acid transport and metabolism.

### 3.4. Whole Genome and Orthologous Gene Comparison

**Figure 4:**
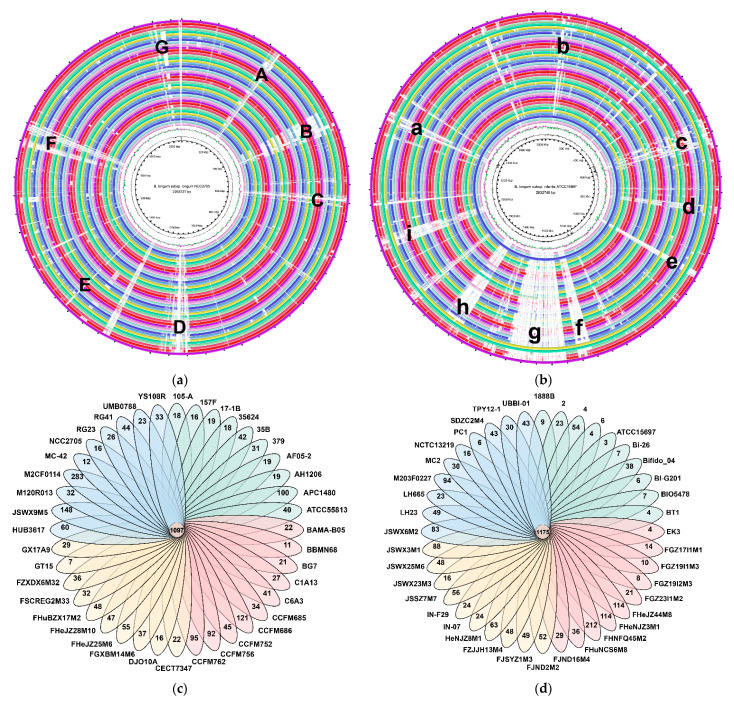
Comparative analysis of *B. longum* subsp. *longum* and *B. longum* subsp. *infantis* genomes: Whole genome sequence comparison of *B. longum* subsp. *longum* (**a**) and *B. longum* subsp. *infantis* (**b**). The blank areas in the figure represent genes that existed in the reference genome but not in other genomes. Orthologous genes and unique genes of *B. longum* subsp. *longum* (**c**) and *B. longum* subsp. *infantis* (**d**).

The whole genomes of *B. longum* subsp. *infantis* and *B. longum* subsp. *longum* were compared via BRIG. *B. longum* subsp. *infantis* ATCC15697 and *B. longum* subsp. *longum* NCC2705 were taken as the reference genomes of each subspecies, respectively. The genomes of *B. longum* subsp. *longum* included seven main regions of variation (Figure 4a); regions A, C and F mainly included the genes related to transport and metabolism of substances, especially carbohydrates; region D mainly included the extracellular polysaccharide synthesis gene clusters; the genes in region B were related to replication, recombination and repair and transcription; region E mainly included the genes related to prophages, transposons and function unknown; and region G consisted of genes related to transcription or defence mechanisms. *B. longum* subsp. *infantis* had more variable regions compared with *B. longum* subsp. *longum* (Figure 4b). Among them, regions a, b, d and h had the same functions as the regions D, C, A and E of *B. longum* subsp. *longum*; region c was mainly related to signal transduction mechanisms; regions e and g contained genes related to replication, recombination and repair; and the main genes in regions f and i had unknown function.

The results of orthologous genes analysis showed that the number of orthologous genes in *B. longum* subsp. *longum* and *B. longum* subsp. *infantis* were 1097 and 1175 (Figure 4c,d), respectively. The difference between orthologous genes of the two subspecies was further compared, and a total of 41 genes only existed in *B. longum* subsp. *infantis*, while there were only ten specific genes for *B. longum* subsp. *longum* (Table S3). Specific genes of *B. longum* subsp. *infantis* mainly included urease gene clusters (BLON_RS00555-BLON_RS00605) and sialic acid metabolism gene clusters (BLON_RS03265-BLON_RS03305) [6], while the specific-genes of *B. longum* subsp. *longum* included transcriptional regulator, transpeptidase and amidohydrolase.

### 3.5. Carbohydrate Utilization Genotype and Phenotype of B. longum subsp. infantis and B. longum subsp. longum

Carbohydrate metabolism was one of the main differences between *B. longum* subsp. *infantis* and *B. longum* subsp. *longum*. In this study, the carbohydrate metabolism phenotype of *B. longum* and its association with genotype were analysed. The prediction of the CAZy database showed that those 80 *B. longum* strains encoded 65 glycosylhydrolase (GH) families and 15 glycosyltransferase (GT) families; in addition, GH19, GH30, GH35, GH43_34, GH50, GH57 and GH13_6 only existed in very few genomes, while GH20, GH2, GH77, GH13_11, GH42, GH3 and GH51 were abundant in *B. longum*.

**Figure 5:**
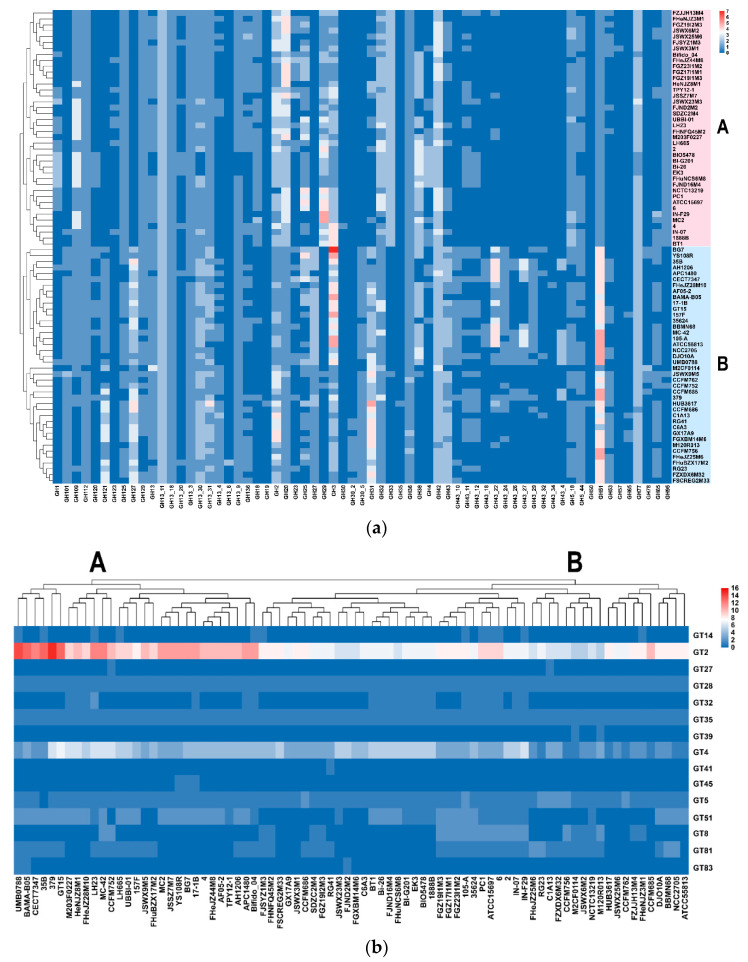

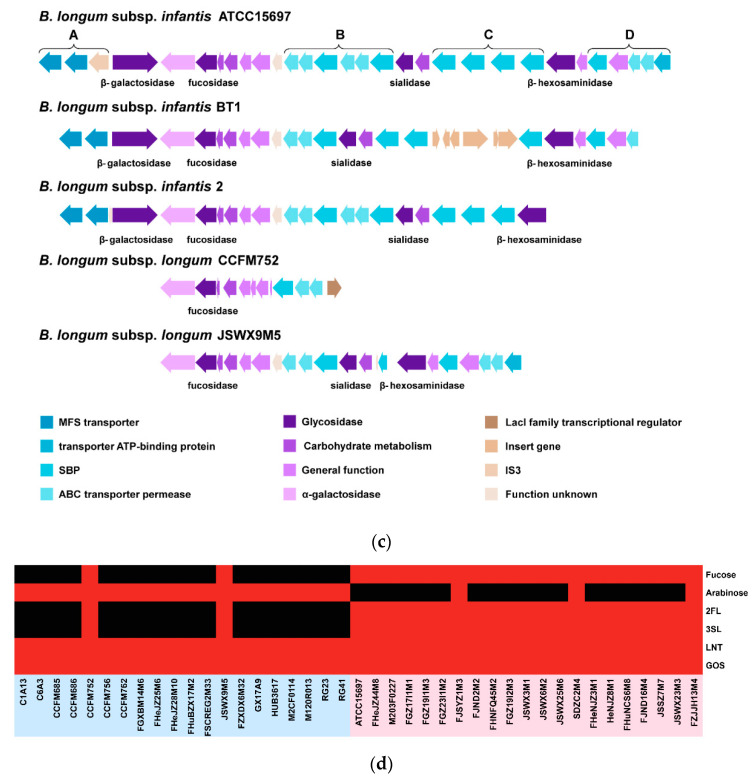
Carbohydrate utilisation genotype and phenotype of *B. longum* subsp. *longum* and *B. longum* subsp. *infantis*: The distribution and number of GH family genes (**a**) and GT family genes (**b**). The number of genes is represented by colour ranging from blue (absent) to red. (**c**) Predicted HMOs utilisation gene cluster in *B. longum*. (**d**) Carbohydrate metabolism capacity of *B. longum*. The horizontal axis represents the strains tested in this study and the vertical axis represents the six carbohydrates used in this test. Red for positive and black for negative. The blue area represents *B. longum* subsp. *longum* and the red area represents *B. longum* subsp. *infantis*.

The cluster analysis of the distribution and abundance of GH families showed that the GH families of *B. longum* could be divided into two groups, in which group A was *B. longum* subsp. *infantis* and group B was *B. longum* subsp. *longum* (Figure 5a). In addition, the significant difference in the number of GH genes between *B. longum* subsp. *infantis* and *B. longum* subsp. *longum* was evaluated by *t*-test, and the results showed that 21 out of 65 GH families were significantly different (*p* < 0.05, data not shown). The GH27, GH121 and GH127 families only existed in *B. longum* subsp. *longum*. Only *B. longum* subsp. *longum* APC1480 lacked the GH27 family; the GH121 family was absent in JSWX9M5, CCFM762, DJO10A and YS108R, while the GH127 family was conserved in all the *B. longum* subsp. *longum* strains. The GH29, GH33 and GH95 families were conserved in all the *B. longum* subsp. *infantis*, and only a few *B. longum* subsp. *longum* strains (such as JSWX9M5 and CCFM752) consisted of those genes. The number of GH43 and its subfamily, GH51, in *B*. *longum* subsp. *infantis* was less than that in *B. longum* subsp. *longum*. In addition, the 15 GT families in *B. longum* could also be divided into two groups (Figure 5b), but there was no significant distinction at the subspecies level (except GT2, *p* < 0.05, data not shown). 

There was a 43kb HMOs utilisation gene cluster in *B. longum* subsp. *infantis* [6]. The BLAST using *B. longum* subsp. *infantis* ATCC15697 (type strain) as a template showed that the four glycosylhydrolase genes in the HMOs utilization gene cluster, including beta-galactosidase (BLON_RS12085), alpha-L-fucosidase (BLON_RS12095), exo-alpha-sialidase (BLON_RS12155) and beta-hexosaminidase (BLON_RS12185), were conserved in all *B. longum* subsp. *infantis*, and the diversities of different strains mainly existed in the four regions (Figure 5c). The number of MFS transporters was different in region A, and a few strains had IS3 and other hypothetical proteins; the difference in region B is the number of ABC transporter permease and ABC transporter substrate-binding proteins. SDZC2M4, FHeNJZ3M1, HeNJZ8M1, BT1, 1888B and IN-07 had inserted genes in region C and the number of SBPs in this region was different; six genes in region D were deleted in JSSZ7M7, FJND2M2, FZJJH13M4, 2, 4 and TPY12-1. Only *B. longum* subsp. *longum* JSWX9M5 and CCFM752 possessed the HMOs utilisation gene clusters similar to that in *B. longum* subsp. *infantis* (Figure 5c). *B. longum* subsp. *longum* JSWX9M5 lacked a beta-galactosidase (BLON_RS12085), and part of SBP compared with *B. longum* subsp. *infantis* ATCC15697, *B. longum* subsp. *longum* CCFM752 had only one L-fucosidase (BLON_RS12095) gene and a linked LacI family transcriptional regulator. Interestingly, there was a beta-galactosidase gene (BLON_RS12085) in most *B. longum* subsp. *longum* strains (except FHuBZX17M2, JSWX9M5 and NCTC13219), but there was no complete gene cluster. In addition, other genes related to HMOs metabolism were found in *B. longum* subsp. *infantis* ATCC15697, including two fucosidase clusters, one sialidase cluster, one beta-galactosidase gene (GH42), and two beta-hexosaminidase (GH20) genes as well as gene clusters related to LNB utilisation (Table 2).

The *B. longum* subsp. *longum* NCC2705 arabinose utilization gene cluster consisted of six genes (Table 3), among which the genes of BL_RS05095, BL_RS05100 and BL_RS05105 were found in all the strains. Genes *araA*, *araB* and *araD* were present in all *B. longum* subsp. *longum* (except CECT7347), but most *B. longum* subsp. *infantis* did not have those three genes. *B. longum* subsp. *infantis* FGZ17I1M1, FGZ19I1M3, FGZ19I2M3 and FGZ23I1M2 only consisted of *araD*, *B. longum* subsp. *infantis* FHNFQ45M2, and JSSZ7M7 only possessed *araB*. Additionally, only *B. longum* subsp. *infantis* SDZC2M4 and FZJJH13M4 had the same arabinose metabolism gene cluster as that in *B. longum* subsp. *longum*. 

The growth of *B. longum* with six carbohydrates as the sole carbon source in vitro was determined. All *B. longum* could grow with GOS and LNT as the sole carbon source. There was a significant difference in the utilisation of the other four carbohydrates between the two subspecies. All the *B. longum* subsp. *infantis* strains were able to grow with fucose as the sole carbon source, while *B. longum* subsp. *longum* could not metabolise it (except CCFM752 and JSWX9M5). Additionally, all the *B. longum* subsp. *longum* strains could use arabinose; by contrast, only a few *B. longum* subsp. *infantis* strains could grow in the presence of arabinose, including FJSYZ1M3, SDZC2M4 and FZJJH13M4. The utilisation of 2’FL and 3’SL by *B. longum* showed that both HMOs supported the growth of all *B. longum* subsp. *infantis*, while the vast majority of *B. longum* subsp. *longum* were not able to metabolise HMOs (except CCFM752 and JSWX9M5) (Figure 5d).

### 3.6. Predicted EPS Gene Clusters in B. longum subsp. infantis and B. longum subsp. longum

**Figure 6:**
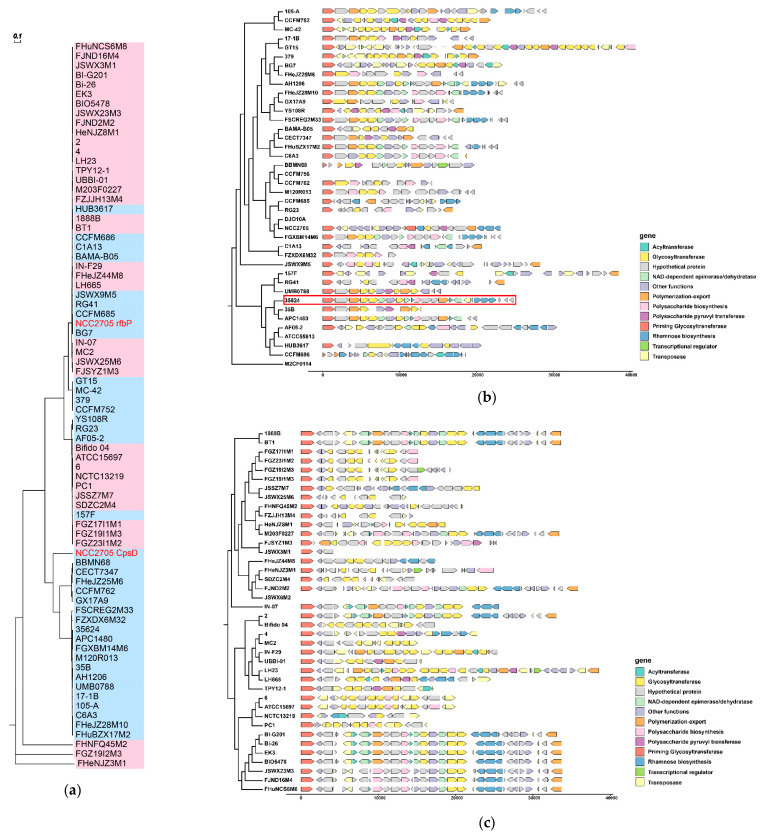
Predicted EPS gene clusters in *B. longum*: (**a**) Phylogenetic tree of *B. longum* based on Priming glycosyltransferase. The blue area represents *B. longum* subsp. *longum*, the red area represents *B. longum* subsp. *infantis* and the red letter highlights two types of p-GTF genes in *B. longum* subsp. *longum* NCC2705. Predicted EPS gene clusters in *B. longum* subsp. *longum* (**b**) and *B. longum* subsp. *infantis* (**c**). The red square highlights the EPS gene clusters, which were used as templates to perform BLAST comparison.

Priming glycosyltransferase (p-GTF) is a key enzyme for synthesizing exopolysaccharides and catalyses the first step in synthesizing many heteropolysaccharides [28]. The genomic analysis of *B. longum* showed that *B. longum* subsp. *longum* ATCC 55813, CCFM756, DJO10A and M2CF0114, as well as *B. longum* subsp. *infantis* JSWX6M2, lacked the p-GTF (*cpsD* or *rfbP*), and only *B. longum* subsp. *longum* NCC2705 had two p-GTFs. The phylogenetic tree based on the genes encoding p-GTF showed that the p-GTF genes of *B. longum* subsp. *infantis* FHNFQ45M2, FGZ19I2M3 and FHeNJZ3M1 were different from other strains. The p-GTF gene in other *B. longum* subsp. *infantis* strains was *rfbP*; while there were two types of p-GTF genes in *B. longum* subsp. *longum* (Figure 6a).

EPS gene clusters defined in two *Bifidobacterium* strains were used as templates to perform BLAST comparison with the remaining *B. longum*, including *B. animalis* subsp. *lactis* DSM 10140, containing most of the genes described in the “consensus” LAB-EPS cluster [29] and *B. longum* subsp. *longum* 35624 [30]. The results showed that the predicted EPS gene clusters were highly variable in different strains, and only some strains showed similar gene cluster composition. For instance, the EPS gene clusters in *B. longum* subsp. *longum* FGXBM14M, FHeJZ28M10, FHuBZX17M2 and FSCREG2M33, as well as *B. longum* subsp. *infantis* FHuNCS6M8, FJND16M4 and JSWX23M3, were relatively similar to that in *B. longum* subsp. *longum* 35624; all the EPS gene clusters in *B. longum* subsp. *infantis* FGZ17I1M1, FGZ19I1M3, FGZ23I1M2 and ATCC15697 contained multiple glycosyltransferases (Figure 6b,c).

### 3.7. CRISPR-Cas Systems in B. longum subsp. infantis and B. longum subsp. longum

**Figure 7:**
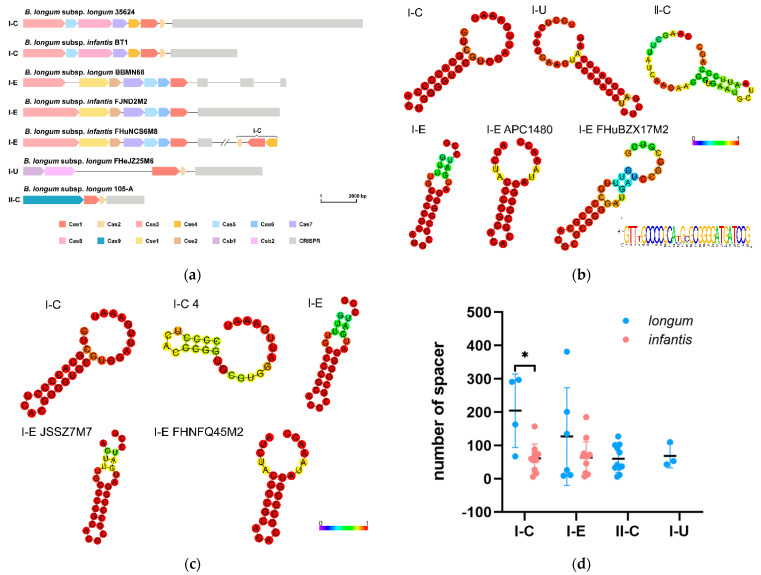
The characteristics of *B. longum* CRISPR: (**a**) CRISPR loci in *B. longum*. (**b**) Predicted RNA secondary structure for repeats of *B. longum* subsp. *longum* and repeat sequences shared by four type I-E strains. The height of the letter indicates the frequency of the corresponding base at that position. (**c**) Predicted RNA secondary structure for repeats of *B. longum* subsp. *infantis*. The minimum free energy (MFE) structure was coloured by base-pairing probabilities. (**d**) Number of *B. longum* spacers. The significant difference was evaluated by *t*-test. * *p* < 0.05.

CRISPR-Cas systems provided adaptive immunity for prokaryotes through DNA-encoded and RNA-mediated nucleic acid targeting mechanisms. *B. longum* CRISPR-Cas systems were predicted by CRISPRCasFinder, and the existence of *cas* genes and the repeats sequence Evidence_Level = 4 in CRISPR loci were used as the basis for evaluating the existence of CRISPR-Cas systems in genomes. The results showed that 25 out of 40 *B. longum* subsp. *longum* strains had a CRISPR-Cas system, including four subtypes I-C, I-E, I-U and II-C, while 24 out of 40 *B. longum* subsp. *infantis* strains had CRISPR-Cas systems with subtypes I-C and I-E (Figure 7a, Table S4). Interestingly, there was another type of I-C Cas protein cluster in some subtypes of I-E in *B. longum* subsp. *infantis*, while *B. longum* subsp. *longum* with subtype I-E did not possess the two types of Cas proteins.

**Figure 8:**
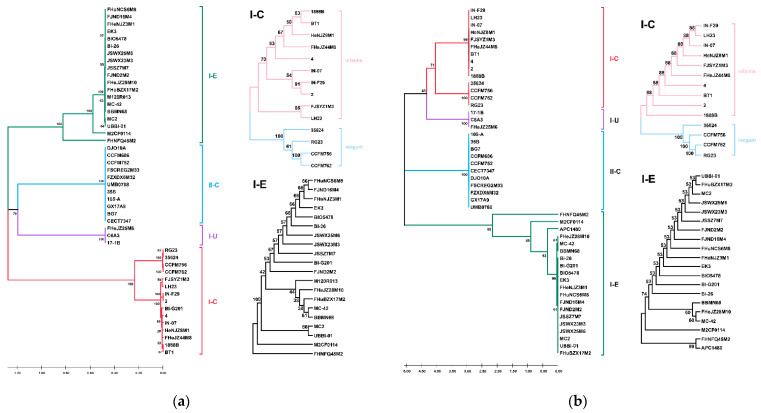
Phylogenetic tree based on Cas1 proteins (**a**) and repeat sequences (**b**). CRISPR-Cas subtypes or subspecies are shown on the right, and each group was marked with colour. The tree was drawn with UPGMA using 1000 bootstrap replicates. Bootstrap values were recorded on the nodes.

The Cas1 protein is the best phylogenetic marker for the evolution of CRISPR-Cas systems. Cas1 protein was present in the CRISPR-Cas systems of all the strains except *B. longum* subsp. *longum* APC1480. The phylogenetic tree, constructed based on the sequence of Cas1 protein, showed that the Cas1 proteins of different subtypes were located on different branches (Figure 8a). The phylogenetic analysis of subtypes I-C and I-E Cas1 proteins showed that the subtype I-C Cas1 proteins of *B. longum* subsp. *infantis* and *B. longum* subsp. *longum* were located on two branches, while the subtype I-E Cas1 protein had no obvious difference between the two subspecies (Figure 8a).

A total of 24 CRISPR were predicted in *B. longum* subsp. *longum* (except M120R013). The length of type I-C repeats was 32 nucleotides and the length of type II-C and I-U repeats was 36 nucleotides, while the length of type I-E repeats was different (Table S4), and the predicted RNA secondary structure was diverse (Figure 7b). There were three variable nucleotides in the 4th, 13th and 15th positions in the repeat sequence of the four type I-E strains, but there was no significant influence on the secondary structure (Figure 7b). A total of 24 CRISPR were predicted in *B. longum* subsp. *infantis*. Except for *B. longum* subsp. *infantis* 4, the type I-C repeat sequence was conserved; the repeat sequences of FHNFQ45M2 and JSSZ7M7 were different from other type I-E strains (Table S4, Figure 7c). Phylogenetic analysis was performed on repeat sequences of *B. longum*, and the results showed that repeat sequences could distinguish the different subtypes of the CRISPR-Cas system in *B. longum* (Figure 8b); repeat sequences of type I-C CRISPR-Cas system could distinguish *B. longum* subsp. *infantis* and *B. longum* subsp. *longum*, while repeat sequences of the type I-E CRISPR-Cas system had no obvious diversity between the two subspecies.

**Figure 9:**
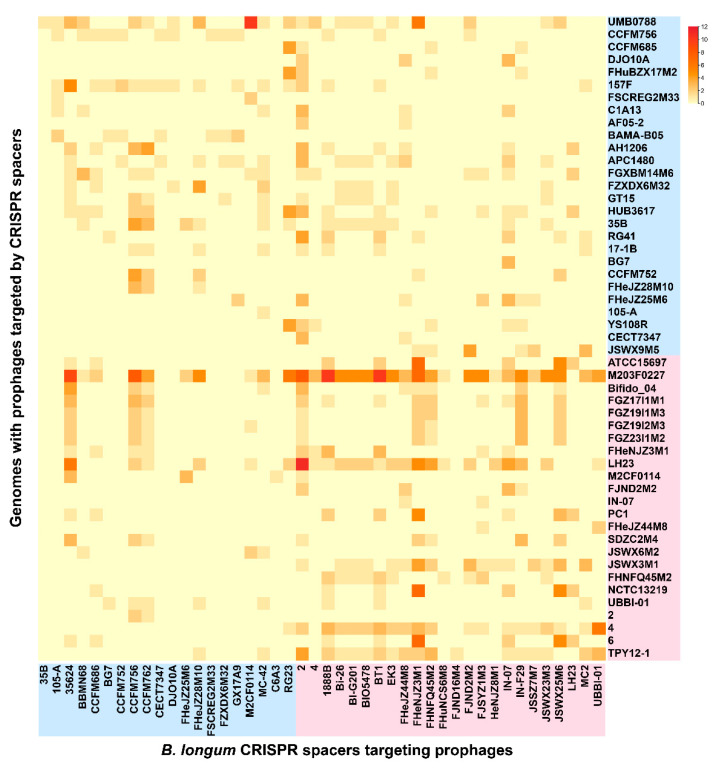
CRISPR spacers targeting prophages in *B. longum*. The vertical axis represented the genomes with prophages targeted by CRISPR spacers. The horizontal axis represents the *B. longum* CRISPR spacers targeting prophages. The heatmap indicates *B. longum* CRISPR spacers that match prophages in the *B. longum* genomes. The number of targeting is represented by colour ranging from yellow (absent) to red. The blue area represents *B. longum* subsp. *longum* and the red area represents *B. longum* subsp. *infantis*.

CRISPR spacers revealed the immune record of the strain and the challenges overcome during DNA invasion. The number of subtypes I-C spacers in *B. longum* subsp. *infantis* was significantly less than that of *B. longum* subsp. *longum* (*p* < 0.05), while there was no significant difference in the number of subtypes I-E spacers between the two subspecies (Figure 7d). Most of the predicted prophages in *B. longum* were incomplete, and intact prophages only existed in *B. longum* subsp. *longum* APC1480 and C1A13 (Table S5). A comparative analysis between the prophages in 80 *B. longum* and the spacers existing in the 48 CRISPR-Cas systems indicated that 24 *B. longum* subsp. *infantis* strains and 20 *B. longum* subsp. *longum* strains possessed at least one spacer which targeted a prophage of *B. longum* (Figure 9). *B. longum* subsp. *longum* spacers matched the prophage in 41 genomes, while the spacers of *B. longum* subsp. *infantis* matched the prophage in 45 genomes. Among *B. longum* subsp. *longum* strains, 35624 possessed the highest number of spacers matching *B. longum* subsp. *longum* 157F, *B. longum* subsp. *infantis* M203F0227 and *B. longum* subsp. *infantis* LH23, while the spacers of *B. longum* subsp. *infantis* 2 and FHeNJZ3M1 matched the *B. longum* prophages by as high as 77 and 67 times, respectively. The prophage of *B. longum* subsp. *infantis* M203F0227 was targeted by *B. longum* spacers the most times, reaching 145 times, and these spacers came from 32 *B. longum*.

### 3.8. Bacteriocin Operons in B. longum subsp. infantis and B. longum subsp. longum

**Figure 10:**
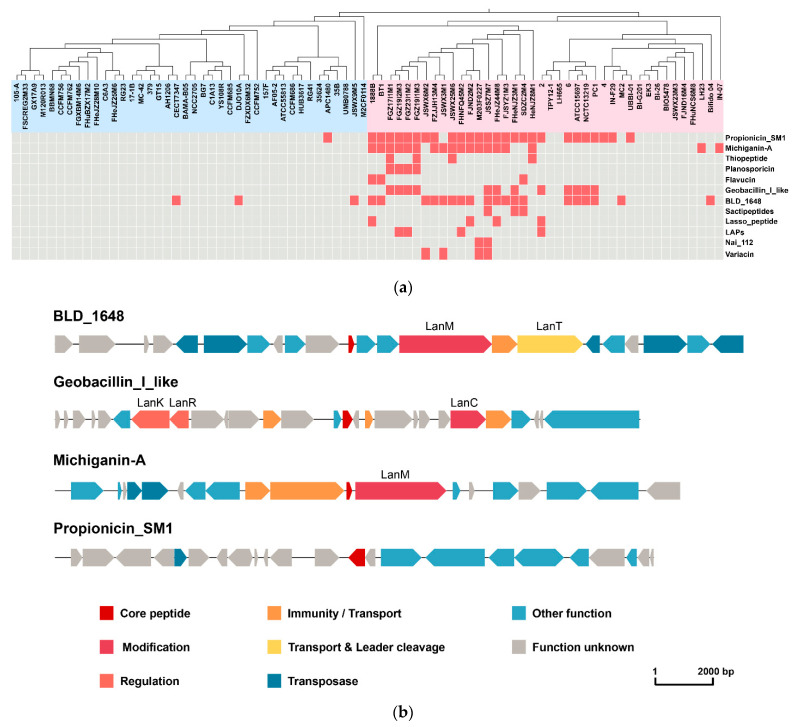
Predicted bacteriocins in *B. longum:* (**a**) Bacteriocins distribution of *B. longum*. The horizontal axis represents the genomes which were clustered according to the phylogenetic tree. The vertical axis represents bacteriocins predicted in *B. longum*. The blue area represents *B. longum* subsp. *longum* and the red area represents *B. longum* subsp. *infantis.* Red indicates present, while grey indicates absents. (**b**) Four predicted bacteriocin structures of *B. longum*.

BAGEL was used to predict the potential bacteriocin operons in *B. longum*, and different types of bacteriocin were distinguished according to the bacteriocin classification method proposed in previous studies. There was no bacteriocin operon in most *B. longum* subsp. *longum* strains; only two bacteriocin operons were predicted in four genomes. A lantibiotic (BLD_1648) was predicted in DJO10A, CECT7347 and JSWX9M5, and there was a class II bacteriocin Propionicin_SM1 (originally isolated from *Propionibacterium jannaschii*) in APC1480 (Table 4, Figure 10a). Differently from *B. longum* subsp. *longum*, there were 12 bacteriocin operons in *B. longum* subsp. *infantis*. All the predicted bacteriocins were class I bacteriocin except for Propionicin_SM1, and seven belonged to class Ia bacteriocin. Only nine *B. longum* subsp. *infantis* strains had no bacteriocin operons. The comparative analysis of the distribution of bacteriocin operons and strains clustering revealed that seven *B. longum* subsp. *infantis* strains without predicted bacteriocin were located on the same branch of the phylogenetic tree (Figure 10a). Additionally, the gene clusters of four bacteriocins widely distributed in *B. longum* were further analysed. The three class I bacteriocins were lanthipeptides, the gene cluster consisting of a gene encoding core peptide, genes encoding a lantibiotic modifying enzyme (LanM), a lantibiotic biosynthesis protein (LanC), a transcriptional regulatory protein (LanR) and genes relating to signal transduction (LanK) and lantibiotic mersacidin transporter(LanT). There was one gene encoding core peptide, one transposase and some genes with other functions in class II bacteriocin Propionicin_SM1 (Figure 10b).

## 4. Discussion

As there are certain differences in phenotypes and genotypes between *B. longum* subsp. *infantis* and *B. longum* subsp. *longum*, such as carbohydrate utilisation, research on *B. longum* should be carried out at the subspecies level; the close genetic relationship makes it difficult for the common methods of species identification to accurately distinguish the two subspecies. Therefore, the classification of *B. longum* subsp. *infantis* and *B. longum* subsp. *longum* is the pre-requisite for comparative genomic analysis. ANI can be used to assess the genetic relationship between subspecies at the genomic level. The results of this study showed that ANI between *B. longum* subsp. *suis* and *B. longum* subsp. *longum*, and that between *B. longum* subsp. *suis* and *B. longum* subsp. *infantis*, was higher than that between *B. longum* subsp. *longum* and *B. longum* subsp. *infantis*, which was consistent with the previous report [8], indicating that the relationship between *B. longum* subsp. *infantis* and *B. longum* subsp. *longum* was relatively distant, and their genetic determinants were different. In addition, the results also showed that ANI among strains of the same subspecies were all greater than 97%, while ANI between different subspecies were all less than 97%. However, the results obtained by different ANI calculation software are slightly different, which may cause classification errors [31], and ANI combined with phylogenetic analysis to distinguish similar species was more reliable. In this study, through further phylogenetic analysis, *B. longum* subsp. *infantis* and *B. longum* subsp. *longum* were distinguished clearly, and there were some misclassified strains in the public database. Previous studies also found similar results, in which a phylogenetic tree was constructed based on 43 selected reference genes and confirmed that *B. longum* subsp. *longum* JDM301 should be *B. longum* subsp. *suis*, while *B. longum* subsp. *infantis* 157F and ATCC 55813 should be *B. longum* subsp. *longum* [8]. The phylogenetic tree constructed based on the core genes of 33 *B. longum* strains also showed errors in the classification of *B. longum* 157F [9]. The comparison of *B. longum* core genes also proved that strain 35624 was indeed *B. longum* subsp. *longum* [30]. *B. longum* subsp. *longum* BXY01 and CMCCP0001 were also reclassified as *B. longum* subsp. *suis* in similar phylogenetic studies [9,11]. Therefore, the classification of different *B. longum* subspecies could be achieved via the combination of ANI and phylogenetic analysis. 

The comparison of general characteristics of *B. longum* genomes showed that there were differences in the size of different genomes. The genome of *B. longum* subsp. *infantis* was generally larger than that of *B. longum* subsp. *longum*, the average GC content of *B. longum* subsp. *infantis* was slightly lower than that of *B. longum* subsp. *longum* and the number of genes in *B. longum* subsp. *infantis* was also more than that in *B. longum* subsp. *longum*. The pan-genome of 23 *B. longum* subsp. *longum* strains has been analysed previously and was confirmed as open, but the number of strains involved was relatively small [8]. Similarly, other pan-genome analyses of *B. longum* contained only a small number of *B. longum* subsp. *infantis* strains [9,11]. The difference between *B. longum* subsp. *infantis* and *B. longum* subsp. *longum* may lead to bias analysis of pan-genome features. Therefore, in this study, the pan-genome of 40 *B. longum* subsp. *infantis* strains and 40 *B. longum* subsp. *longum* strains was analysed and compared. The results showed that the pan-genome of *B. longum* subsp. *infantis* and *B. longum* subsp. *longum* was nearly closed, which indicated that, with the addition of new strains, new genes would appear in the genomes of the two subspecies, but the probability of new genes appearing is low.

The comparison of whole genome sequence revealed the differences in the intraspecies genetic information of *B. longum* subsp. *longum* and *B. longum* subsp. *infantis*. The results showed that different genomes of *B. longum* subsp. *infantis* had greater diversities than those of *B. longum* subsp. *longum*. Strains of both subspecies had variability in carbohydrate transport and metabolism genes and EPS gene clusters. The comparison of homologous gene diversities revealed the interspecific differences between the two subspecies. The number of specific genes in *B. longum* subsp. *infantis* was four times than that in *B. longum* subsp. *longum*, and it mainly included two larger gene clusters. This may be related to the utilisation of breast milk by *B. longum* subsp. *infantis*. The urease gene cluster (BLON_RS00555-BLON_RS00605) was related to the utilisation of urea in breast milk. As the protein concentration in breast milk often makes it difficult to meet the increasing nitrogen demand during the growth of infants, the urea in breast milk becomes another potential nitrogen sources for infants and their gut microbes; bacterial urease (EC5.3.1.5) plays a major role in urea nitrogen recovery (UNS) [6]. Comparative genomic hybridization analysis of 15 *B. longum* strains has been performed in a previous study, and the results indicated that genes and their activity of urea metabolism were only conserved in *B. longum* subsp. *infantis* [32]. The sialic acid metabolism gene cluster (BLON_RS03265-BLON_RS03305) is related to the metabolism of HMOs. Although this gene cluster did not exist in *B. longum* subsp. *longum* strains in this study, it was found that ten *B. longum* subsp. *longum* strains isolated from younger subjects contained the genes encoding sialidase homologous to that in *B. longum* subsp. *infantis* ATCC 15697 via gene enrichment [33]. The existence of specific genes in *B. longum* subsp. *infantis* may further explain why *B. longum* subsp. *infantis* are more widely present in the intestine of breast-fed infants.

The proportion of annotated gene encoding enzymes involved in complex carbohydrates metabolism were more than 8% in the *B. longum* genome [9,34]. GTs and GHs are responsible for the synthesis of glycosidic bonds and hydrolysis (or modification), respectively, and are the two major enzymes related to metabolising carbohydrates. In this study, the GH and GT families of *B. longum* were compared and the results showed that *B. longum* subsp. *infantis* and *B. longum* subsp. *longum* had significant differences in the composition of GHs, while GTs showed no difference at the subspecies level. The GH27 family contained various enzymes such as α-galactosidase and β-L-arabinopyranosidase, the GH121 family mainly encoded β-L-arabinobiosidase, the GH127 family contained β-L-arabinofuranosidases and the GH43 family mainly included α-L-arabinanase, β-xylosidase, L-arabinofuranosidase and other enzymes related to the metabolism of complex plant-derived polysaccharides. Moreover, the GH29, GH33 and GH95 families were related to HMOs utilization. Therefore, there were gene families related to the utilisation of HMOs in *B. longum* subsp. *infantis*, while the number of gene families related to the utilisation of plant-derived polysaccharides in *B. longum* subsp. *longum* was greater, which was consistent with the previous reports [8,9].

In earlier studies, it was found that *B. longum* subsp. *infantis* can grow well in a medium with HMOs as the sole carbon source, while *B. longum* subsp. *longum* could not utilise HMOs or had weak utilisation ability [35,36,37], but the latest research has found that certain *B. longum* subsp. *longum* strains could also utilise 2’FL and 3’FL in HMOs [38,39]. Therefore, the metabolism-related genes of the three HMOs, 2’FL, 3’SL and LNT, were analysed and further determined the metabolic ability of *B. longum*. Both *B. longum* subsp. *longum* and *B. longum* subsp. *infantis* could metabolise LNT (Galβ 1-3GlcNAc linkage) similar to previous reports [38,40]. LNT was decomposed into LNB and lactose under the action of β-hexosaminidases [6], and a gene encoding β-hexosaminidases and genes related to LNB metabolism were present in both *B. longum* subsp. *infantis* and *B. longum* subsp. *longum*. Additionally, beta-galactosidases (BLON_RS10470) was also related to LNT metabolism [41], which explains the reason for *B. longum* subsp. *longum* metabolising LNT. All *B. longum* subsp. *infantis* strains in this study could metabolise the three HMOs assayed. The deletion of some transporters and the insertion of gene fragments in the HMO utilisation gene cluster did not affect the metabolic ability of *B. longum* subsp. *infantis* on 2’FL and 3’SL. The presence of partial HMO utilisation gene clusters in *B. longum* subsp. *longum* JSWX9M5 and CCFM752 enabled them to metabolise 2’FL and 3’SL. The HMO utilisation gene cluster in *B. longum* subsp. *longum* JSWX9M5 was more similar to that in *B. longum* subsp. *infantis* ATCC15697, while the HMO utilisation gene cluster in *B. longum* subsp. *longum* CCFM752 was consistent with that found in *B. longum* subsp. *longum* SC596 [38]. Interestingly, *B. longum* subsp. *longum* CCFM752 could also metabolise 3’SL, although there was no presence of sialidase in its genome.

Previous studies have shown that *B. longum* subsp. *infantis* could use L-fucose, but *B. longum* subsp. *longum* could not [42]; hence, L-arabinose was considered to be a potential marker distinguishing *B. longum* subsp. *longum* and *B. longum* subsp. *infantis* [7]. It was found that a permease and a fucosidase in *B. longum* subsp. *infantis* ATCC15697 seemed to replace the three arabinose metabolism genes (BL_RS05080-BL_RS05090) in *B. longum* subsp. *longum* NCC2705 [43]. No fucosidase clusters and fucose metabolism ability were found in other *B. longum* subsp. *longum* strains, except for JSWX9M5 and CCFM752. It was found that there was an arabinose metabolism gene cluster in *B. longum* subsp. *infantis* SDZC2M4 and FZJJH13M4, and in vitro growth studies also proved that both the two strains could metabolise arabinose. It is worth noting that an arabinose gene cluster was not found in the genome of *B. longum* subsp. *infantis* FJSYZ1M3. Those studies showed that the differences in carbohydrate metabolism-related genes determined the different carbohydrate metabolism capabilities of strains, and further revealed that *B. longum* subsp. *infantis* preferred to metabolise HMOs, while *B. longum* subsp. *longum* had a stronger utilisation capacity for plant-derived carbohydrates. Kujawska et al. analysed the *B. longum* isolated from infant faecal samples from 1 to 18 months and found that the difference in carbohydrate metabolism between *B. longum* subsp. *infantis* and *B. longum* subsp. *longum* was compatible with the changes in the infant’s diet, particularly the transition from breast milk to a more diverse diet [44], which could further explain why the niches of *B. longum* subsp. *longum* and *B. longum* subsp. *infantis* were different.

Previous studies have shown that the exopolysaccharides produced by *B. longum* were involved in the immune regulation functions [45,46]. Therefore, the EPS gene clusters in *B. longum* were predicted in this study. There was no p-GTF in a few genomes. Phylogenetic analysis showed that p-GTF in different strains had high homology, which may be due to the existence of conserved domains involved in the interaction with lipid carriers [29]. A previous work used the EPS gene cluster in *B. animalis* subsp. *lactis* DSM10140 as a template to predict the EPS gene cluster in *B. longum* subsp. *longum* NCC2705 and *B. longum* subsp. *infantis* ATCC15697, and found that EPS gene clusters of the two strains were quite different; among which, there were more glycosyltransferases in *B. longum* subsp. *infantis* ATCC15697, while genes encoding rhamnose biosynthetic precursors only existed in the EPS gene cluster of *B. longum* subsp. *longum* NCC2705 [29]. Altmann et al. focused on the EPS gene cluster and EPS structure of *B. longum* subsp. *longum* 35624 and found that only some *B. longum* subsp. *longum* strains showed partial similarity with its EPS gene cluster. The EPS gene clusters of *Bifidobacterium* have great variability, both interspecific and intraspecific, which created uncertainty in the prediction of the EPS gene cluster in *B. longum* subsp. *longum* and *B. longum* subsp. *infantis*. This study predicted the EPS gene clusters of *B. longum* subsp. *longum* and *B. longum* subsp. *infantis* and found diversity between different strains, but there was no significant difference between the two subspecies. Because the exopolysaccharide biosynthesis in *Bifidobacterium* is still unclear, the prediction of its gene cluster is mainly based on sequence homology studies, and the correlation between the *B. longum* EPS gene cluster and its physicochemical properties cannot be determined [29]. For example, *B. longum* subsp. *longum* CCFM756 and M2CF0114 in this study did not have a complete EPS gene cluster, but they were still able to produce EPS [47]. Therefore, the association analysis of *B. longum* EPS production and its gene cluster still needs further investigation. 

CRISPR-Cas systems provide an adaptive genetic resistance mechanism for prokaryotes. In this study, we analysed the existence and diversity of CRISPR-Cas systems in *B. longum* subsp. *infantis* and *B. longum* subsp. *longum*. Previous research on *B. longum* CRISPR-Cas systems found that the types of CRISPR-Cas system in *B. longum* subsp. *infantis* and *B. longum* subsp. *longum* were different, and subtypes II-C and I-U only existed in *B. longum* subsp. *longum* [10], which was consistent with our results, although many strains in the study classified as *B. longum* subsp. *infantis* previously should have been *B. longum* subsp. *longum.* In addition, in the phylogenetic analysis of Cas1 proteins and repeat sequences of type I-C and I-E CRISPR-Cas systems, it was found that Cas1 protein and repeat sequences of subtype I-C *B. longum* subsp. *longum* and *B. longum* subsp. *infantis* were significantly different, which may be used as a basis for distinguishing *B. longum* subsp. *longum* and *B. longum* subsp. *infantis*. Previous studies found that a large number of *B. longum* spacers matched the prophages of other species, indicating that these species lived in the same niches, where co-evolution occurred between the CRISPR-Cas system and the prophage [10]. In this study, spacers in *B. longum* subsp. *infantis* targeted the prophages of *B. longum* subsp. *infantis* more than *B. longum* subsp. *longum* prophages, and *B. longum* subsp. *longum* spacers also targeted *B. longum* subsp. *longum* prophages more frequently. This seemed to be able to further explain that *B. longum* subsp. *infantis* and *B. longum* subsp. *longum* niches were similar, but there were certain differences; therefore, *B. longum* subsp. *infantis* mainly exists in the intestine of breast-fed infants, while *B. longum* subsp. *longum* is widely present in the intestine of humans of different ages.

Bacteriocin is a kind of antimicrobial peptide synthesised by bacterial ribosomes. It usually acts by inducing pore formation in target cells or inhibiting the synthesis of cell walls. It has been considered as a potential substitute for antibiotics in the future [48]. Among the bacteriocins of *B. longum* identified by BAGEL4, only BLD_1648 was isolated from *Bifidobacterium*, while other bacteriocins were originally isolated from other species, such as *Propionibacterium jensenii*, *Geobacillus kaustophilus* and *Clavibacter michiganensis*. This may be due to the fact that only a few bifidobacterial bacteriocins have been purified and identified in previous studies. Bacteriocins BLD_1648 from *B. longum* subsp. *longum* DJO10A is a lantibiotic with antimicrobial activity against both Gram-positive bacteria and Gram-negative bacteria. It has been deeply characterised, and the amino acid sequence of it has at least been partially elucidated [49,50]. The prediction results of this study showed that bacteriocin operons in *B. longum* were class I bacteriocin, except for Propionicin_SM1. Previous studies have found that *B. bifidum* NCFB 1454 could produce bifidocin B, which belongs to class IIa bacteriocin with strong antilisterial activity [50]. However, no bacteriocins of *B. longum* homologous to bifidocin B were found in this study. In addition, bacteriocins were more widespread in *B. longum* subsp. *infantis* compared with *B. longum* subsp. *longum*, which might reveal the stronger antimicrobial activity and greater competitive advantage of *B. longum* subsp. *infantis*. Although this study found potential bacteriocin operons in *B. longum* subsp. *infantis*, relying on in silico screening, the production of its functional bacteriocins needs further experimental verification.

## 5. Conclusions

In this study, 40 genomes of *B. longum* subsp. *infantis* and 40 genomes of *B. longum* subsp. *longum* were compared via comparative genomics analyses. The results revealed the differences between the two subspecies mainly existed in carbohydrate utilisation, CRISPR-Cas systems and bacteriocin operons, as well as the diversity of EPS gene clusters. This study expanded the understanding of differences in the genomic characteristics of *B. longum* subsp. *infantis* and *B. longum* subsp. *longum*, and provided references for the further development and application of *B. longum* probiotic resources.

## Figures and Tables

**Table 1:** The information of 80 *B. longum* strains.

Strain	Genome Size (Mbp)	GC (%)	CDS Number	Origin	Accession Number
2	2.62	59.40	2075	Human Faeces	SAMEA5770183
4	2.57	59.60	2053	Human Faeces	SAMEA5770185
6	2.83	59.90	2405	Human Faeces	SAMEA5770187
105-A	2.29	60.10	1770	Human Faeces	SAMD00019943
157F	2.41	60.11	1929	Infant Faeces	SAMD00060953
17-1B	2.47	60.20	1959	Human Faeces	SAMN02862993
1888B	2.58	59.40	2039	Infant Faeces	SAMN06621708
35624	2.26	60.00	1758	Human Faeces	SAMN04254466
35B	2.51	60.10	1967	Human Faeces	SAMN00829158
379	2.39	60.20	1902	Human Faeces	SAMN04155602
AF05-2	2.43	60.40	1978	Human Faeces	SAMN09734186
AH1206	2.42	60.20	1955	Infant Faeces	SAMN04576213
APC1480	2.48	59.90	2017	Human Faeces	SAMN07958358
ATCC15697	2.83	59.90	2411	Infant Faeces	SAMN02598380
ATCC55813	2.40	60.10	1901	Infant Faeces	SAMN00001475
BAMA-B05	2.27	59.90	1800	Human Faeces	SAMN12569298
BBMN68	2.27	59.90	1747	Human Faeces	SAMN02603469
BG7	2.46	60.01	1926	Infant Faeces	SAMN03271682
Bi-26	2.61	59.30	2078	Infant Faeces	SAMN10380491
Bifido_04	2.58	59.70	2110	Human Blood	SAMEA51817918
BI-G201	2.57	59.30	2028	Human Faeces	SAMN14908987
BIO5478	2.57	59.40	2093	Infant Faeces	SAMN12856543
BT1	2.58	59.40	2030	Infant Faeces	SAMN03271683
C1A13	2.31	59.86	2000	Human Faeces	SAMN19128425
C6A3	2.40	60.03	2037	Human Faeces	SAMN16976870
CCFM685	2.35	59.69	2025	Infant Faeces	SAMN16976872
CCFM686	2.50	59.76	2125	Infant Faeces	SAMN19128426
CCFM752	2.28	59.70	1939	Infant Faeces	SAMN17575072
CCFM756	2.34	60.00	2075	Infant Faeces	SAMN16976873
CCFM762	2.40	59.89	2037	Infant Faeces	SAMN19128427
CECT7347	2.33	60.00	1869	Unknown	SAMEA3146249
DJO10A	2.39	60.12	1874	Unknown	SAMN02603512
EK3	2.56	59.40	2061	Human Faeces	SAMN02862995
FGXBM14M6	2.52	60.15	2262	Human Faeces	SAMN16976885
FGZ17I1M1	2.72	59.60	2583	Infant Faeces	SAMN16976996
FGZ19I1M3	2.66	59.66	2506	Infant Faeces	SAMN16976991
FGZ19I2M3	2.72	59.60	2574	Infant Faeces	SAMN16976986
FGZ23I1M2	2.72	59.60	2597	Infant Faeces	SAMN16976987
FHeJZ25M6	2.50	59.96	2150	Human Faeces	SAMN19128429
FHeJZ28M10	2.39	59.99	2033	Human Faeces	SAMN19128430
FHeJZ44M8	2.72	59.22	2551	Infant Faeces	SAMN16976998
FHeNJZ3M1	2.71	59.70	2599	Infant Faeces	SAMN16976982
FHNFQ45M2	2.84	59.57	2781	Infant Faeces	SAMN16976993
FHuBZX17M2	2.43	60.14	2122	Human Faeces	SAMN19128431
FHuNCS6M8	2.55	59.40	2332	Infant Faeces	SAMN16976988
FJND16M4	2.56	59.39	2390	Infant Faeces	SAMN16976985
FJND2M2	2.54	59.51	2296	Infant Faeces	SAMN16976992
FJSYZ1M3	2.63	59.70	2441	Infant Faeces	SAMN16976990
FSCREG2M33	2.36	60.16	2030	Human Faeces	SAMN16976909
FZJJH13M4	2.57	59.62	2336	Infant Faeces	SAMN19128438
FZXDX6M32	2.31	59.77	1961	Human Faeces	SAMN16976921
GT15	2.34	60.00	1815	Human Faeces	SAMN03093230
GX17A9	2.49	59.88	2072	Human Faeces	SAMN16976922
HeNJZ8M1	2.67	59.55	2447	Infant Faeces	SAMN16976984
HUB3617	2.46	59.94	2175	Human Faeces	SAMN19128434
IN-07	2.75	60.00	2189	Human Faeces	SAMD00047616
IN-F29	2.64	59.90	2109	Human Faeces	SAMD00047617
JSSZ7M7	2.62	59.35	2330	Infant Faeces	SAMN16976979
JSWX23M3	2.56	59.38	2311	Infant Faeces	SAMN19128437
JSWX25M6	2.62	59.55	2372	Infant Faeces	SAMN16976983
JSWX3M1	2.63	58.89	2306	Infant Faeces	SAMN16976989
JSWX6M2	2.72	59.92	2569	Infant Faeces	SAMN16976980
JSWX9M5	2.54	60.04	2276	Infant Faeces	SAMN19128439
LH23	2.76	59.60	2293	Infant Faeces	SAMEA4838174
LH665	2.59	59.40	2087	Infant Faeces	SAMEA4838176
M120R013	2.20	60.02	1897	Human Faeces	SAMN16976924
M203F0227	2.77	59.61	2639	Infant Faeces	SAMN16976997
M2CF0114	2.37	59.77	2085	Human Faeces	SAMN19128435
MC2	2.56	59.60	2011	Human Faeces	SAMEA5574696
MC-42	2.29	59.80	1775	Infant Faeces	SAMN04263942
NCC2705	2.26	60.11	1769	Infant Faeces	SAMN02603675
NCTC13219	2.60	60.00	2235	Unknown	SAMEA104318167
PC1	2.79	59.80	2387	Human Faeces	SAMEA51825418
RG23	2.26	60.02	1911	Human Faeces	SAMN16976925
RG41	2.49	60.25	2147	Human Faeces	SAMN19128436
SDZC2M4	2.73	59.64	2586	Infant Faeces	SAMN16976981
TPY12-1	2.64	59.70	2128	Infant Faeces	SAMN05578879
UBBI-01	2.73	59.40	2234	Fermented Food	SAMN11370925
UMB0788	2.45	60.20	2012	Female Urinary Tract	SAMN08193649
YS108R	2.52	60.10	2021	Human Faeces	SAMN09355369

**Table 2:** Predicted HMOs metabolism-related genes of *B. longum*.

Enzymes/Carbohydrates	Gene (Cluster)	*B. longum* subsp. *longum*	*B. longum* subsp. *infantis*
Fucosidase	BLON_RS01290- BLON_RS01310	Absent	Present (except FHeNJZ3M1, SDZC2M4)
Fucosidase	BLON_RS02195-BLON_RS02205	Absent	Present (except FHeNJZ3M1, SDZC2M4)
Sialidases	BLON_RS03255-BLON_RS03305	Absent	Present
beta-galactosidases	BLON_RS10470	Present	Present
N-acetyl-β-D-hexosaminidases	BLON_RS02365	Absent (except JSWX9M5)	Present (except LH665)
N-acetyl-β-D-hexosaminidases	BLON_RS03705	Present	Present
LNB	BLON_RS11215- BLON_RS11245	Present (except M2CF0114)	Present

**Table 3:** Arabinose metabolism gene clusters of *B. longum* subsp. *longum*.

Gene ID	Gene Name	Protein ID	Annotation
BL_RS05080	araA	WP_007051388.1	L-arabinose isomerase
BL_RS05085	araB	WP_007051389.1	L-ribulose-5-phosphate 4-epimerase
BL_RS05090	araD	WP_007051390.1	FGGY-family carbohydrate kinase
BL_RS05095	-	WP_008782880.1	LacI family DNA-binding transcriptional regulator
BL_RS05100	-	WP_011068401.1	ribonuclease HII
BL_RS05105	lepB	WP_010081174.1	signal peptidase I

**Table 4:** Class and sources of predicted bacteriocins in *B. longum*.

Bacteriocin	B. *longum* subsp. *longum*	B. *longum* subsp. *infantis*	Class	Sources
Propionicin_SM1	1	26	II	*Propionibacterium jensenii*
Michiganin-A	-	18	Ia, Lanthipeptide B	*Clavibacter michiganensis* subsp. *michiganensis*
Geobacillin_I_like	-	12	Ia, Lanthipeptide A	*Geobacillus kaustophilus* HTA426
BLD_1648	3	19	Ia, Lanthipeptide B	*B. longum* subsp. *longum* DJO10A
Propeptin_2	-	4	If, Lasso_peptide	*Microbispora* sp. SNA-115
Nai_112	-	2	Ia, Lanthipeptide C	*Actinoplanes* sp. NAI112
Variacin	-	4	Ia, Lanthipeptide B	*kocuria varians*
Planosporicin	-	4	Ia, Lanthipeptide A	*Microbispora* sp. 107891
Flavucin	-	3	Ia, Lanthipeptide A	*Corynebacterium lipophiloflavum*
Thiopeptide	-	4	Thiopeptide	-
LAPs	-	5	Id, LAPs	-
Sactipeptides	-	3	Ic, Sactipeptides	-

## Data Availability

All raw sequencing data analysed in this study have been submitted to the NCBI Sequence Read Archive (https://www.ncbi.nlm.nih.gov/sra/) (accessed on 20 July 2021) under the BioProject PRJNA681061, PRJNA730421, PRJNA732070 and PRJNA694834.

## Supplementary Materials

The following are available online at https://www.mdpi.com/article/10.3390/microorganisms9081713/s1, Table S1: The information of *B. longum* subsp. *suis*, Table S2: Average nucleotide identity (ANI) values of *B. longum*, Table S3: Specific genes for *B. longum* subsp. *infantis* or *B. longum* subsp. *longum*, Table S4: CRISPR-Cas systems in *B. longum*, Table S5: Predicted prophages in *B. longum*.

## References

[1] Mattarelli P., Bonaparte C., Pot B., Biavati B. (2008). Proposal to reclassify the three biotypes of *Bifidobacterium longum* as three subspecies: *Bifidobacterium longum* subsp. *longum* subsp. nov., *Bifidobacterium longum* subsp. infantis comb. nov. and *Bifidobacterium longum* subsp. suis comb. nov. Int. J. Syst. Evol. Microbiol..

[2] Matteuzzi D., Crociani F., Zani O., Trovatelli L.D. (1971). *Bifidobacterium* suis n. sp.: A new species of the genus *Bifidobacterium* isolated from pig faces. J. Basic Microbiol..

[3] Groeger D., O’Mahony L., Murphy E.F., Bourke J.F., Dinan T., Kiely B., Shanahan F., Quigley E.M. (2013). *Bifidobacterium infantis* 35624 modulates host inflammatory processes beyond the gut. Gut Microbes.

[4] Miyauchi E., Ogita T., Miyamoto J., Kawamoto S., Morita H., Ohno H., Suzuki T., Tanabe S. (2013). *Bifidobacterium longum* alleviates dextran sulfate sodium-induced colitis by suppressing il-17a response: Involvement of intestinal epithelial costimulatory molecules. PLoS ONE.

[5] Barba-Vidal E., Castillejos L., Colom P.L., Urgell M.R., Muñoz J.A.M., Martín-Orúe S.M. (2017). Evaluation of the probiotic strain *Bifidobacterium longum* subsp. Infantis CECT 7210 capacities to improve health status and fight digestive pathogens in a piglet model. Front. Microbiol..

[6] Sela D.A., Chapman J., Adeuya A., Kim J., Chen F., Whitehead T.R., Lapidus A., Rokhsar D., Lebrilla C.B., German J.B. (2008). The genome sequence of *Bifidobacterium longum* subsp. infantis reveals adaptations for milk utilization within the infant microbiome. Proc. Natl. Acad. Sci. USA.

[7] Sakata S. (2002). Unification of *Bifidobacterium* infantis and *Bifidobacterium* suis as *Bifidobacterium longum*. Int. J. Syst. Evol. Microbiol..

[8] Chaplin A.V., Efimov B.A., Smeianov V., Kafarskaia L.I., Pikina A.P., Shkoporov A. (2015). Intraspecies genomic diversity and long-term persistence of *Bifidobacterium longum*. PLoS ONE.

[9] O’Callaghan A., Bottacini F., Motherway M.O., Van Sinderen D. (2015). Pangenome analysis of *Bifidobacterium longum* and site-directed mutagenesis through by-pass of restriction-modification systems. BMC Genom..

[10] Hidalgo-Cantabrana C., Crawley A., Sanchez B., Barrangou R. (2017). Characterization and exploitation of CRISPR loci in *Bifidobacterium longum*. Front. Microbiol..

[11] Arboleya S., Bottacini F., O’Connell-Motherway M., Ryan C.A., Ross R.P., Van Sinderen D., Stanton C. (2018). Gene-trait matching across the *Bifidobacterium longum* pan-genome reveals considerable diversity in carbohydrate catabolism among human infant strains. BMC Genom..

[12] Jiang J., Yang B., Ross R., Stanton C., Zhao J., Zhang H., Chen W. (2020). Comparative genomics of pediococcus pentosaceus isolated from different niches reveals genetic diversity in carbohydrate metabolism and immune system. Front. Microbiol..

[13] Richter M., Rosselló-Móra R. (2009). Shifting the genomic gold standard for the prokaryotic species definition. Proc. Natl. Acad. Sci. USA.

[14] Chen C., Chen H., Zhang Y., Thomas H.R., Frank M.H., He Y., Xia R. (2020). TBtools: An integrative toolkit developed for interactive analyses of big biological data. Mol. Plant.

[15] Chen F. (2006). OrthoMCL-DB: Querying a comprehensive multi-species collection of ortholog groups. Nucleic Acids Res..

[16] Katoh K., Standley D.M. (2016). A simple method to control over-alignment in the MAFFT multiple sequence alignment program. Bioinformatics.

[17] Subramanian B., Gao S., Lercher M.J., Hu S., Chen W.-H. (2019). Evolview v3: A webserver for visualization, annotation, and management of phylogenetic trees. Nucleic Acids Res..

[18] Zhao Y., Wu J., Yang J., Sun S., Xiao J., Yu J. (2012). PGAP: Pan-genomes analysis pipeline. Bioinformatics.

[19] Alikhan N.-F., Petty N.K., Ben Zakour N.L., Beatson S.A. (2011). BLAST Ring Image Generator (BRIG): Simple prokaryote genome comparisons. BMC Genom..

[20] Lombard V., Ramulu H.G., Drula E., Coutinho P.M., Henrissat B. (2014). The carbohydrate-active enzymes database (CAZy) in 2013. Nucleic Acids Res..

[21] Couvin D., Bernheim A., Toffano-Nioche C., Touchon M., Michalik J., Néron B., Rocha E.P.C., Vergnaud G., Gautheret D., Pourcel C. (2018). CRISPRCasFinder, an update of CRISRFinder, includes a portable version, enhanced performance and integrates search for cas proteins. Nucleic Acids Res..

[22] Kumar S., Stecher G., Li M., Knyaz C., Tamura K. (2018). MEGA X: Molecular evolutionary genetics analysis across computing platforms. Mol. Biol. Evol..

[23] Lorenz R., Bernhart S.H.F., Zu Siederdissen C.H., Tafer H., Flamm C., Stadler P.F., Hofacker I.L. (2011). ViennaRNA Package 2.0. Algorithms Mol. Biol..

[24] Crooks G.E., Hon G., Chandonia J.-M., Brenner S.E. (2004). WebLogo: A sequence logo generator. Genome Res..

[25] Arndt D., Grant J.R., Marcu A., Sajed T., Pon A., Liang Y., Wishart D.S. (2016). PHASTER: A better, faster version of the PHAST phage search tool. Nucleic Acids Res..

[26] Van Heel A.J., De Jong A., Song C., Viel J., Kok J., Kuipers O.P. (2018). BAGEL4: A user-friendly web server to thoroughly mine RiPPs and bacteriocins. Nucleic Acids Res..

[27] Lugli G.A., Milani C., Turroni F., Duranti S., Ferrario C., Viappiani A., Mancabelli L., Mangifesta M., Taminiau B., Delcenserie V. (2014). Investigation of the evolutionary development of the genus *Bifidobacterium* by comparative genomics. Appl. Environ. Microbiol..

[28] Ruas-Madiedo P., Moreno J.A., Salazar N., Delgado S., Mayo B., Margolles A., Reyes-Gavilán C.G.D.L. (2007). Screening of exopolysaccharide-producing lactobacillus and *Bifidobacterium* strains isolated from the human intestinal microbiota. Appl. Environ. Microbiol..

[29] Hidalgo-Cantabrana C., Sánchez B., Milani C., Ventura M., Margolles A., Ruas-Madiedo P. (2014). Genomic overview and biological functions of exopolysaccharide biosynthesis in *Bifidobacterium* spp.. Appl. Environ. Microbiol..

[30] Altmann F., Kosma P., O’Callaghan A., Leahy S., Bottacini F., Molloy E., Plattner S., Schiavi E., Gleinser M., Groeger D. (2016). Genome analysis and characterisation of the exopolysaccharide produced by *Bifidobacterium longum* subsp. *longum* 35624™. PLoS ONE.

[31] Figueras M.J., Beaz-Hidalgo R., Hossain M.J., Liles M.R. (2014). Taxonomic affiliation of new genomes should be verified using average nucleotide identity and multilocus phylogenetic analysis. Genome Announc..

[32] LoCascio R.G., Desai P., Sela D.A., Weimer B., Mills D.A. (2010). Broad conservation of milk utilization genes in *Bifidobacterium longum* subsp. infantis as revealed by comparative genomic hybridization. Appl. Environ. Microbiol..

[33] Odamaki T., Bottacini F., Kato K., Mitsuyama E., Yoshida K., Horigome A., Xiao J.-Z., Van Sinderen D. (2018). Genomic diversity and distribution of *Bifidobacterium longum* subsp. *longum* across the human lifespan. Sci. Rep..

[34] Ventura M., Canchaya C., Tauch A., Chandra G., Fitzgerald G.F., Chater K.F., van Sinderen D. (2007). Genomics of actinobacteria: Tracing the evolutionary history of an ancient phylum. Microbiol. Mol. Biol. Rev..

[35] Locascio R.G., Niñonuevo M.R., Kronewitter S.R., Freeman S.L., German J.B., Lebrilla C.B., Mills D.A. (2008). A versatile and scalable strategy for glycoprofiling *Bifidobacterial* consumption of human milk oligosaccharides. Microb. Biotechnol..

[36] Asakuma S., Hatakeyama E., Urashima T., Yoshida E., Katayama T., Yamamoto K., Kumagai H., Ashida H., Hirose J., Kitaoka M. (2011). Physiology of consumption of human milk oligosaccharides by infant gut-associated *Bifidobacteria*. J. Biol. Chem..

[37] Garrido D., Ruiz-Moyano S., Lemay D., Sela D.A., German J.B., Mills D.A. (2015). Comparative transcriptomics reveals key differences in the response to milk oligosaccharides of infant gut-associated *Bifidobacteria*. Sci. Rep..

[38] Garrido D., Ruiz-Moyano S., Kirmiz N., Davis J.C., Totten S.M., Lemay D., Ugalde J.A., German J.B., Lebrilla C.B., Mills D.A. (2016). A novel gene cluster allows preferential utilization of fucosylated milk oligosaccharides in *Bifidobacterium longum* subsp. *longum* SC596. Sci. Rep..

[39] Bunesova V., Lacroix C., Schwab C. (2016). Fucosyllactose and L-fucose utilization of infant *Bifidobacterium longum* and *Bifidobacterium* kashiwanohense. BMC Microbiol..

[40] Lawley B., Centanni M., Watanabe J., Sims I., Carnachan S., Broadbent R., Lee P.S., Wong K.H., Tannock G.W. (2018). Tuf gene sequence variation in *Bifidobacterium longum* subsp. infantis detected in the fecal microbiota of Chinese infants. Appl. Environ. Microbiol..

[41] Yoshida E., Sakurama H., Kiyohara M., Nakajima M., Kitaoka M., Ashida H., Hirose J., Katayama T., Yamamoto K., Kumagai H. (2012). *Bifidobacterium longum* subsp. infantis uses two different β-galactosidases for selectively degrading type-1 and type-2 human milk oligosaccharides. Glycobiology.

[42] Crociani F., Alessandrini A., Mucci M.M., Biavati B. (1994). Degradation of complex carbohydrates by *Bifidobacterium* spp.. Int. J. Food Microbiol..

[43] Sela D.A., Mills D.A. (2010). Nursing our microbiota: Molecular linkages between *Bifidobacteria* and milk oligosaccharides. Trends Microbiol..

[44] Kujawska M., La Rosa S.L., Roger L.C., Pope P.B., Hoyles L., McCartney A.L., Hall L.J. (2020). Succession of *Bifidobacterium longum* strains in response to a changing early life nutritional environment reveals dietary substrate adaptations. iScience.

[45] Schiavi E., Gleinser M., Molloy E., Groeger D., Frei R., Ferstl R., Rodriguez-Perez N., Ziegler M., Grant R., Moriarty T.F. (2016). The surface-associated exopolysaccharide of *Bifidobacterium longum* 35624 plays an essential role in dampening host proinflammatory responses and repressing local T_H_17 responses. Appl. Environ. Microbiol..

[46] Yan S., Yang B., Zhao J., Zhao J., Stanton C., Ross R., Zhang H., Chen W. (2019). A ropy exopolysaccharide producing strain *Bifidobacterium longum* subsp. *longum* YS108R alleviates DSS-induced colitis by maintenance of the mucosal barrier and gut microbiota modulation. Food Funct..

[47] Yan S., Zhao G., Liu X., Zhao J., Zhang H., Chen W. (2017). Production of exopolysaccharide by *Bifidobacterium longum* isolated from elderly and infant feces and analysis of priming glycosyltransferase genes. RSC Adv..

[48] Cotter P.D., Ross R., Hill C. (2013). Bacteriocins—A viable alternative to antibiotics?. Nat. Rev. Genet..

[49] Lee J.-H., Li X., O’Sullivan D.J. (2011). Transcription analysis of a lantibiotic gene cluster from *Bifidobacterium longum* DJO10A. Appl. Environ. Microbiol..

[50] Martinez F.C., Balciunas E.M., Converti A., Cotter P.D., Oliveira R.P.D.S. (2013). Bacteriocin production by *Bifidobacterium* spp. A review. Biotechnol. Adv..

